# Learning Laparoscopic Radical Hysterectomy: Are We Facing an Emerging Situation?

**DOI:** 10.3390/ijerph20032053

**Published:** 2023-01-22

**Authors:** Graziella Moufawad, Antonio Simone Laganà, Nassir Habib, Vito Chiantera, Andrea Giannini, Federico Ferrari, Amerigo Vitagliano, Luigi Della Corte, Giuseppe Bifulco, Zaki Sleiman

**Affiliations:** 1 Department of Obstetrics and Gynecology, Lebanese American University Medical Center-Rizk Hospital, Beirut 1100, Lebanon; 2 Unit of Gynecologic Oncology, ARNAS “Civico—Di Cristina—Benfratelli”, Department of Health Promotion, Mother and Child Care, Internal Medicine and Medical Specialties (PROMISE), University of Palermo, 90127 Palermo, Italy; 3 Obstetrics and Gynecology Department, Francois Quesnay Hospital, 78200 Mantes-La-Jolie, France; 4 Department of Medical and Surgical Sciences and Translational Medicine, PhD Course in “Translational Medicine and Oncology”, Sapienza University, 00185 Rome, Italy; 5 Department of Clinical and Experimental Sciences, University of Brescia, 25123 Brescia, Italy; 6 Division of Obstetrics and Gynecology, ASST Spedali Civili di Brescia, 25123 Brescia, Italy; 7 Unit of Obstetrics and Gynecology, Department of Biomedical and Human Oncologic Science, University of Bari, 70100 Bari, Italy; 8 Department of Neuroscience, Reproductive Sciences and Dentistry, School of Medicine, University of Naples Federico II, 80138 Naples, Italy; 9 Department of Public Health, University of Naples Federico II, 80138 Naples, Italy

**Keywords:** cervical cancer, abdominal radical hysterectomy, laparoscopic radical hysterectomy, robotic radical hysterectomy, learning curve, LACC trial

## Abstract

Despite wide screening campaigns and early detection, cervical cancer remains the fourth most common cancer among women. Radical hysterectomy, whether by open, laparoscopic or by robotic-assisted techniques, is the mainstay treatment. However, for adequate surgical results and good oncological prognosis, a gynecological surgeon should be trained to perform those procedures. The learning curve of radical hysterectomy, especially by laparoscopy, is influenced by several factors. The LACC trial, the decrease in cervical cancer incidence and radical hysterectomy procedures have widely reduced the learning curve for surgeons. This article mainly discusses the learning curve of laparoscopic radical hysterectomy for cervical cancers, and how several factors are influencing it negatively, with the need to have medical authorities reset specific surgical training programs and allocate them to special oncological centers.

## 1. Introduction

Cervical cancer is the fourth most common cancer among women worldwide [1]. The mainstay treatment is radical hysterectomy for early-stage cervical cancer. For over 100 years, open radical hysterectomy has been used, since it was first described by Wertheim [2]. Later, in the early 1990s, techniques for laparoscopic radical hysterectomy and lymph node dissection were developed, and considerable new experience has been gained since then, with more than 1000 cases being reported in the literature [3].

As with the integration of any surgical procedure, a learning curve is an important part of assessment of the procedure and its efficiency [1]. Thus, studying the learning curve of radical hysterectomy for early-stage cervical cancer is of utmost importance. Several studies have been conducted to evaluate the learning curve of surgeons in gynecological oncological procedures [4], and these studies were important to assess and compare a surgeon’s expertise and improvement.

In this review, we present a brief overview of the learning curve of radical hysterectomy for cervical cancer by laparotomy and laparoscopy. We also discuss factors that might be influencing this learning curve, such as the major effect of the LACC trial and declining incidence of radical hysterectomies.

## 2. Materials and Methods

This review was conducted according to the Preferred Reporting Items for Systematic Reviews and Meta-Analyses (PRISMA) Statement [5], to clearly identify the learning curve of surgeons in radical hysterectomy for cervical cancer, via different surgical routes. The effects of the declining incidence of radical hysterectomies and the LACC trial were also thoroughly investigated during our search. The review was registered in PROSPERO (CRD42022383094) before starting the search. We used “Pubmed” and “Google Scholar” as our search engines. Medical Subject Heading terms and keywords used were ((radical hysterectomy) AND (cervical cancer)) AND ((learning curve) AND (laparoscopy OR celioscopy OR laparotomy OR abdominal OR robotic-assisted OR robotics); also searched keywords included (LACC trial), (radical hysterectomy incidence), and (cervical cancer). Our search was complemented via screening references of retrieved articles and the studies included in previous reviews on the topic. A focus on the presentation of the learning curve of laparoscopic radical hysterectomy was our main point of interest, together with factors influencing it, such as the LACC trial and the decreasing number of the radical hysterectomies. We screened observational studies, case reports, case series, and randomized controlled trials. To better identify studies needed for our systematic review, two authors (G.M., N.H.) evaluated articles retrieved via our search strategy, by screening titles and abstracts. The full texts of these potentially eligible articles were retrieved and independently assessed for eligibility by two other review team members (A.S.L., A.G.). Any disagreement between them over the eligibility of particular articles was resolved through discussion with an external collaborator.

**Figure 1:**
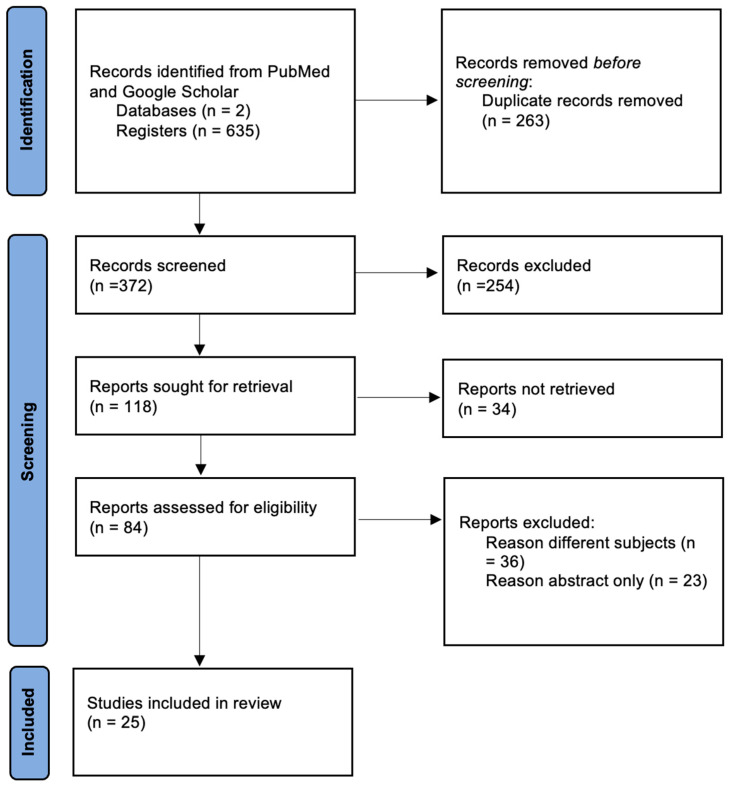
Preferred Reporting Items for Systematic Reviews and Meta-Analyses (PRISMA) flowchart for study screening, selection, and inclusion.

As summarized in Figure 1, 635 records were found, from which 263 duplicates were removed. A total of 372 records were left to be screened. After screening the abstract, 254 records were further excluded, and 118 reports were sought for retrieval. Of these, 34 reports were not retrieved. A total of 84 reports were assessed for eligibility, and 25 studies were included in our review. Inclusion criteria included articles discussing the learning curve of radical hysterectomy for cervical cancer using the three aforementioned techniques: laparoscopy, laparotomy, and robotic-assisted techniques. Articles studying the LACC trial and its effects on radical hysterectomy routes were included. Articles discussing the learning curve of surgeons for indications other than cervical cancer or for operations other than radical hysterectomy were excluded. Additional articles discussing cervical cancer screening, incidence, and the associated incidence of radical hysterectomy were also included. Study characteristics such as populations, methods, and results were extracted by two independent authors (L.D.C. and F.F.) using an already set standard to decrease discrepancy. When inconsistency ensued, a third external collaborator helped resolve the issue via discussion. We chose a narrative synthesis of the results, which are summarized in Table 1 and further discussed throughout.

## 3. Radical Hysterectomy Learning Curve: In Numbers and Percentages

A learning curve, or the number of procedures required to achieve proficiency, is important during the integration of any new procedure [25]. Surgeons may develop surgical proficiency after only a few cases, and the number of cases required varies depending on the type of surgery performed, the training center, training surgeons, and other factors.

In a retrospective study conducted by Kim et al., 89 patients with early-stage cervical cancer underwent radical hysterectomy via open, laparoscopic, or robotic techniques [1]. Learning curves of each type of surgery were obtained, and the minimum numbers of cases required to achieve surgical improvement were 16 in radical abdominal hysterectomy, 13 in laparoscopic radical hysterectomy, and 21 in robotic-assisted radical hysterectomy. This study concluded that surgical proficiency could significantly affect the surgical outcome in minimally invasive surgery.

Another study was conducted by Li et al. to study the impact of learning curve on the survival of abdominal or minimally invasive radical hysterectomy in early-stage cervical cancer. Thirty cases were required to achieve an acceptable 5 year disease-free survival rate in the open hysterectomy group versus 60 cases in the laparoscopic group [6]. The authors also concluded that the surgeons’ proficiency affected survival in both groups when tumors were ≥2 cm. Anchora et al. concluded that a surgeon’s experience is an independent prognostic factor in minimally invasive radical hysterectomy for early-stage cervical cancer; indeed, a steady reduction in disease recurrence was directly correlated to surgeons’ experience [7].

A study was conducted by Reade et al. to study the learning curve of laparoscopic radical hysterectomy after its implementation in 2007 at Hamilton Health Sciences institution. This study concluded that 23 cases were needed to improve a surgeon’s proficiency, and this relatively low number might have been due to “buddy operating” [9]. They described the new technique “buddy operating” where two surgeons combine their skills for better results, a novel technique important for reducing the learning curve for infrequently performed complex procedures. Similarly, Kong et al. [9] found that gynecologic oncologists could reach a level of surgical proficiency only after 20 cases of laparoscopic radical hysterectomy, even without any previous abdominal radical hysterectomy experience. Regarding robotic radical hysterectomy, Yim et al. [11] concluded in their study that surgical proficiency can be achieved after 28 cases.

In a retrospective analysis conducted by Liu et al. [10] on patients who received radical hysterectomy for stage IB cervical cancer from 2001 to 2015, no substantial survival improvement was achieved between the years 2001 and 2015 following the adoption of the laparoscopic method. This could be explained by the learning curve of laparoscopic radical hysterectomy. Thus, this article implies that laparoscopic radical hysterectomy needs extensive training, and its learning curve could hinder a surgeon’s performance and affect the results. 

Regarding laparoscopic radical hysterectomy with lymph node dissection for cervical cancer, a study was performed by Hwang et al. [12] to evaluate the learning curve and to compare the surgico-pathologic outcomes in patients treated during the beginning of the learning curve with patients treated in the second half of the curve. The authors concluded that an extended learning period was required for laparoscopic radical hysterectomy with lymph node dissection; however, survival and pathologic outcomes were not affected during this extended period. Oladikun et al. [13] conducted an audit on the radical hysterectomies performed in their center in Nigeria for cervical carcinoma from 2006 to 2008. They concluded that, despite limited resources in developing countries, definitive surgery for early cervical cancer is feasible. With respect to the learning curve of surgeons, an audit of surgical care of surgical cancer can help improve a surgeon’s skills. 

To study the learning curve of laparoscopic radical hysterectomy with or without pelvic lymphadenectomy, Chong et al. [14] conducted a retrospective review and divided patients into two groups: the first 50 cases who underwent laparoscopic radical hysterectomy and the second 50 cases. According to this study, surgical outcome was improved with experience, and the complication rate in group 1 was higher than that in group 2. However, there was no significant difference in survival between those two groups.

With respect to robotic-assisted radical hysterectomy, Schreuder et al. [2] concluded that the introduction of robotic-assisted technique requires a learning curve of fewer than 15 cases, which would reduce the operating time to a level less than open surgery. In another study conducted by Heo et al. [15] to study the learning curve of robotic radical hysterectomy with node dissection, it was found that a minimum of 13 cases were required to achieve surgical improvement in the treatment of cervical cancer

## 4. Is the Number of Radical Hysterectomies Performed Decreasing?

A large study was conducted by Lycke et al. [18], which included 98,484 patients in a nationwide population-based study using national Danish registries between the years 2000 and 2015. Incidence rate of hysterectomy declined over time, which was mainly due to a decline in the rate of benign hysterectomy, also probably due to wide HPV vaccination campaigns and early screening [27,28]. Similarly, a shift in surgical procedure was observed over time form abdominal to minimally invasive surgical procedure. 

In a review conducted by Schaafsma et al. [25], the authors was concluded that there was a shift toward less radical treatment for early-stage cervical cancer. Indeed, new treatment strategies have been developed recently in order to reduce surgical morbidity without affecting oncological safety.

A review published by Undurraga et al. [20] discussed appropriate management for early-stage cervical cancer. Individualization of treatment to reduce side-effects of therapy and to improve quality of life has led to using prognostic factors in allocating patients to either radical surgery, “less radical” surgery, or radiotherapy. Conservative surgery for cervical cancer was described by Sheperd et al. [29]. They concluded that, due to extensive screening, most cases of cervical cancer are diagnosed in reproductive-age women who stress on fertility-preserving methods. Thus, more uterine-conserving and less radical surgeries have been performed, which might affect the learning curve of surgeons.

## 5. The LACC Trial: Did It Change Surgeons’ Attitude?

The Laparoscopic Approach for Cervical Cancer (LACC) trial revealed that radical hysterectomy in women with early-stage cervical cancer is associated with higher rates of disease-free survival and overall survival in the open-abdominal technique compared to the laparoscopic technique [24]. 

Lewiki et al. [26] investigated 2437 patients to study the effect of the LACC trial on minimally invasive surgery, and a substantial reduction in the use of minimally invasive surgery for surgical cancer was found in academic and nonacademic centers after the publication of the LACC trial.

According to Eoh et al., one of the most important prognostic factors for robotic-assisted radical hysterectomy for cervical cancer is the operation year, which represents the institutional learning curve [8]. In a study conducted by Qin et al. [16], the authors found that laparoscopic radical hysterectomy was not inferior to abdominal radical hysterectomy if performed by a single team with adequate laparoscopic experience. Thus, surgical experience greatly affects oncological outcome; from here arises the importance of a learning curve, factors that can influence or ameliorate learning curves, and the importance of taking learning curves into consideration in the training of residents and fellows.

## 6. Discussion

A learning curve with a minimum of several cases is required to acquire the necessary surgical skills for radical hysterectomy whether by open technique or by laparoscopy. Several of the studies mentioned above stressed on the superior outcomes seen after more practice and more experience. According to Chong et al. [14], patients treated during the second part of the learning curve had fewer complications and better overall outcomes. 

Working as a team in a referral center while operating together improves learning curve and, hence, the surgical outcome. Reade et al. [19] described the term “buddy operating”, where two surgeons combine referrals and operate together, thereby achieving faster skill acquisition. This, indeed, is very important as it shortens the learning curve and provides better surgical outcomes with fewer complications and faster skill acquisition for the surgeons. Hence, operating in referral centers rather than general hospitals, where the team is more specialized and has more experience and skills, would help improve a new surgeon’s learning curve and provide them with a faster and more efficient way of learning.

However, due to the extensive screening and early diagnosis, medical management is becoming the norm, thus decreasing the number of operations, which was also affected by the pandemic [30,31,32], thus making it more difficult for current residents and fellows to improve their learning curve.

On another note, it was found that the number of radical hysterectomies is decreasing due to extensive screening, wide vaccination campaigns, and earlier diagnosis or even fertility-preservation reasons [33,34].

The LACC trial was published in November 2018, finding disease-free survival and overall survival to be higher in open radical hysterectomy rather than minimally invasive radical hysterectomy. Following the publication of these results, gynecological surgery for cervical cancer switched toward a laparotomic approach in order to avoid worse oncological outcomes. From this perspective, although some small retrospective analyses reported comparable surgical outcomes of abdominal radical hysterectomy and total laparoscopic radical hysterectomy [21], the quality of the evidence from the LACC trial is superior, and this led the most authoritative international societies, such as the European Society of Gynecologic Oncology (ESGO), to recommend not to use minimal invasive surgery, unless in a prospective trial. In addition, we should differentiate between squamous cervical cancer (which was prevalent in the LACC trial) and other histotypes which may have different outcomes according to the surgical approach [35]. Lastly, accumulating evidence suggests that oncological outcomes of laparoscopic radical hysterectomy can be influenced by preoperative conization [23,36].

The LACC trial definitely had a negative effect on the learning curve of radical hysterectomy. Overall, we shifted from open to minimally invasive surgery with all expenses on the learning curve and surgeons’ performance; now, we will have to shift back to open surgery if the LACC trial is widely applied.

It has been already established that a surgeon’s experience impacts the oncological outcomes in patients with early-stage cervical cancer [3]. According to Anchora et al. [7], the scientific community should establish the minimum training required in the field of minimally invasive radical hysterectomy for early-stage cervical cancer. 

Thus, operative management of cervical cancer should be performed in a referral center where a minimum number of yearly cases is performed for better outcomes, with appropriate case selection [17] to improve the learning curve.

## 7. Conclusions

To conclude, radical hysterectomy is definitely a procedure declining in numbers due to extensive screening and early detection [37,38]. Surgical proficiency requires a minimum number of cases performed before becoming autonomous; thus, a learning curve is getting more and more difficult to achieve, especially regarding systematic lymph-node aortic staging [22]. For residents and fellows, graduating without achieving this learning curve and performing laparoscopic radical hysterectomy will not only lead to complications, but also affect the oncological process and, thus, survival.

Do the medical authorities in different countries need to newly propose a different pattern for training or allocate specific centers with special programs for training for laparoscopic radical hysterectomy? This is an emerging situation that definitely needs to be addressed in the near future.

## Figures and Tables

**Table 1:** Main characteristics and outcomes of the included studies.

Authors, Year	Patients	Open or Minimally Invasive	Oncological Outcome	Morbidity for the Early Years of the Learning Curve	Morbidity for the Late Years of the Learning Curve
Kim et al., 2021 [1]	89	Open, robotic-assisted, and minimally invasive	Surgical proficiency could significantly affect oncological outcome	Poorer progression-free survival	Improved progression free survival and survival rates
Li et al., 2021 [6]	280	Open and minimally invasive	5 year disease-free and overall survival rates	NA	NA
Pedone Anchora et al., 2021 [7]	243	Minimally invasive	3-year disease-free survival	75.4%	91.6%
Eoh et al., 2020 [8]	310	Robotic-assisted and open techniques	Progression-free survival and overall survival	NA	Operation year decreased morbidity in the late years of the learning curve
Kong et al., 2015 [9]	84	Minimally invasive	Operating time	Longer operating time	Shorter operating time
Liu et al., 2019 [10]	406	Open and minimally invasive	5-year disease-free survival	NA	5-year disease-free survival increased with years
Yim et al., 2013 [11]	65	Minimally invasive	Blood loss and early post operative complications	225 mL blood loss, 28% postop complications	100 mL blood loss, 8.1% postop complications
Hwang et al., 2012 [12]	70	Minimally invasive	Mean operating time, complication rate	307 min, N = 9	266 min, N = 1
Oladokun et al., 2010 [13]	10	Abdominal	Operative time and blood loss		Linear reduction in surgical blood loss and operative time
Chong et al., 2009 [14]	100	Minimally invasive	Operative time, length of hospital stay, transfusion rate		Significant decrease in operative time, length of hospital stay, and transfusion rate
Schreuder et al., 2010 [2]	28	Abdominal and robotic assisted	Operative time	9 h	4 h
Heo et al., 2018 [15]	41	Robotic-assisted and laparoscopic	Average operation time	Longer	Shorter
Qin et al., 2020 [16]	256	Abdominal and minimally invasive	Progression-free survival and overall survival	Similar rates	Similar rates
Yaribakht et al., 2015 [4]	72	Robotic-assisted	Surgeon console time	Increased surgeon’s console time	Decreased surgeon’s console time
Zakashansky et al., 2008 [3]	NA	Abdominal, laparoscopic-assisted, and robotic-assisted	Safety profile, blood loss, hospital stay	NA	Improved safety profile and blood loss with laparoscopic surgery
Kim et al., 2015 [17]	161	Laparoscopic-assisted	Surgical and survival outcomes	Longer operating time, more intraoperative ureter injury	Lesser blood loss and shorter post op hospital stay
Lycke et al., 2021 [18]	98,484	Abdominal and minimally invasive surgery	NA	NA	NA
Reade et al., 2011 [19]	45	Laparoscopic-assisted	Operative time, estimated blood loss, number of lymph nodes removed, hospital stay	201 min, 355 mL, N = 11.5, 1.57 days	176 min, 196 mL, N = 15.3, 0.14 days
Undurraga et al., 2010 [20]	NA	Laparoscopy	Complication rate, quality of life	NA	NA
Pecorino et al., 2022 [21]	196	Abdominal and laparoscopic-assisted	Operative time, estimated blood loss, hospital stay, transfusions	NA	NA
Di Donna et al., 2022 [22]	1200	Laparoscopic and robotic-assisted	Estimated blood loss, hospital stay	NA	Estimated blood loss was higher in laparoscopy compared with robotic surgery, and hospital stay was longer in robotic assisted surgery compared with laparoscopic surgery
Kim et al., 2022 [23]	578	Abdominal and laparoscopic-assisted	Disease-free survival	NA	NA
Ramirez et al., 2018 [24]	631	Abdominal and minimally invasive surgery	Disease-free survival and overall survival	NA	NA
Schaafsma et al., 2022 [25]	NA	Abdominal and minimally invasive surgery	NA	NA	NA
Lewicki et al., 2021 [26]	2437	Abdominal and minimally invasive surgery	Number of minimally invasive surgeries performed after LACC trial was published	NA	NA

NA: not available.

## Data Availability

The data described in this article were retrieved by previously published studies.
